# Italian Validation of the Capacity to Love Inventory: Preliminary Results

**DOI:** 10.3389/fpsyg.2018.01434

**Published:** 2018-08-15

**Authors:** Giorgia Margherita, Anna Gargiulo, Gina Troisi, Francesca Tessitore, Nestor D. Kapusta

**Affiliations:** ^1^Department of Humanities, University of Naples Federico II, Naples, Italy; ^2^Department of Psychoanalysis and Psychotherapy, Medical University of Vienna, Vienna, Austria

**Keywords:** capacity to love, capacity to love inventory, psychodynamic perspective, Italian validation, psychometric properties

## Abstract

**Introduction:** Within a wider international research project aimed at operationalize the psychodynamic construct of capacity to love (Kernberg, 2011), the Capacity to Love Inventory (CTL-I) is a 41-items self-report questionnaire assessing six dimensions: interest in the life project of the other, basic trust, gratitude, common ego ideal, permanence of sexual passion, loss, and mourning.

**Objectives:** The study is aimed at validating the Italian version of the CTL-I.

**Method:** A total sample of 736 Italian non-clinical adults was administered a checklist assessing socio-demographic variables, and the CTL-I. A Confirmatory Factor Analyses (CFA) was conducted to examine the construct validity of the Italian version of the CTL-I. Only a part of the total sample (320 participants) was administered an additional series of concurrent measures in order to investigate the convergent validity of the CTL-I. Correlations with measures of socio-sexual orientation, quality of romance relations, and psychopathological questionnaires were examined through Pearson's correlation coefficients.

**Results:** CFA results suggested that the Italian CTL-I fully replicated the six-factor structure of the original CTL-I. Cronbach's alpha index provided satisfactory results for all subscales and the correlations with concurrent measures were in expected direction.

**Conclusion:** The results showed promising psychometric characteristics of the Italian version of CTL-I. Implications of the feasibility of the instrument in clinical and psychotherapeutic settings are discussed.

## Introduction

Starting from the Kernberg psychoanalytic theory (1995), the capacity to love is a human being's disposition to establish relationships with others and it is tightly connected to a person's psychic development. Thus, mature love, characterized by a set of dimensions, is considered as a theoretical frame with diagnostic potentialities. In fact, the incapacity to fall in love could be an important diagnostic marker in clinical contexts. For example, as highlighted by Kernberg (1974), narcissistic personalities have serious distortions in their internal relationships with others, demonstrating an incapacity to fall in love.

Previous studies have explored the associations between romantic relationships and depression highlighting that relationships of depressive people are characterized by greater dependency, emotional neediness and anger (Matussek et al., 1986; Fiske and Peterson, 1991). Moreover, negative qualities of romantic relationships seem have an important role in predicting depressive symptoms (La Greca and Harrison, 2005).

Building on the theory of capacity to love (Kernberg, 2011), Kapusta et al. (2018) have developed the Capacity to Love Inventory, which consists of 41 items with six dimensions (interest in the other, basic trust, gratitude, common ego ideal, permanence of sexual passion, loss, and mourning), with a good internal consistency in each subscale.

In the Italian context, instruments assessing love relationships have been developed from various theoretical perspectives: the attachment perspective (Gentili et al., 2002; Marazziti et al., 2010; Busonera et al., 2014; Carli et al., 2016), the developmental perspective (Ponti et al., 2010) and the psychosocial one (Donato et al., 2009; Boffo and Mannarini, 2015). The CTL-I adds a psychodynamic perspective into research of love relations and introduces new dimensions involved in romantic love.

The present study aims at validating the Italian version of the CTL-I (Kapusta et al., 2018) using a CFA methodology in an Italian sample. To assess the convergent validity of the CTL-I, correlations with some measures were examined through Pearson's correlation coefficients.

## Method

### Participants

The total sample was composed of 736 Italian non-clinical adults (488 women and 248 men). Mean age of the participants was 28.6 years (*SD* = 9.8).

The only inclusion criterion was the age of 18 and higher. The presence of conditions affecting the possibility of taking the assessment or the refusal of consent form was considered as exclusion criteria. Four hundred and sixteen participants were recruited through advertisements (*flyers* and newspapers distributed at the university and at public events). Before starting the surveys, participants signed a written consent form to participate the research. Instead, the remaining 320 participants were recruited through online *ads* posted at established community groups and through mailing lists. The online recruited subsample was asked to read a web page with the informed consent document and to accept it by clicking a button in order to start the research and complete the online survey. The whole study protocol was approved by the ethics committee of section of Psychology and Educational Sciences, University of Naples Federico II.

All the participants were administered the CTL-I. Out of the total of participants, only participants recruited through the online survey (320 participants: 249 women and 71 men) were administered a series of concurrent measures of depression, narcissism, socio-sexual orientation, quality of romance relations. Mean age of this subsample was 31.9 years (*SD* = 11.08). The relationship status of participants was as follows: 79.7% in a relationship, 17.8% single, 1.3% married, 1.3% divorced (Table 1).

**Table 1:** Characteristics of the sample (*n* = 736).

**Variables**	**Percentages (Mean ± SD)**
**SEX**	
Men	248
Women	488
Age – M(SD)	(28.6 ± 9.8)
**EDUCATION LEVEL**	
Primary school	0.3%
Secondary school	5.8%
High school	37.5%
Graduated	51.3%
Post-graduate degree	0.5%
**RELATIONSHIP STATUS**	
In a relationship	65.7%
Single	28.2%
Married	3.7%
Divorced	0.8%
Widowed	0.7%

### Data analysis procedure

All the analyses were performed with the Statistical Package for the Social Sciences (SPSS) 19.0 and MPLUS7 software (Bentler, 1990; Schermelleh-Engel et al., 2003; Muthén and Muthén, 2012).

Confirmatory factor analysis on the Italian version of CTL-I was performed using the Maximum Likelihood (ML) as appropriate estimator.

We tested the theory driven model developed by Kapusta et al. (2018) by means of a CFA: a six-factor model with 41 items and scales being allowed to correlate with each other, in line with Kapusta et al. (2018) results. Model fit was assessed by means of the following fit indexes: (1) the chi-squared (χ2) statistic and its degree of freedom; (2) the Standardized Root Mean Square Residuals (SRMR); and, (3) the Root Mean Square Error of Approximation (RMSEA) and its 90% confidence interval (90% CI). In line with what Schermelleh-Engel et al. (2003) affirmed, the model fit the data when x2/df equal or <2, RMSEA equal or <0.05 (90% CI: the lower boundary of the CI should contain zero for exact fit and be < 0.05 for close fit), although Browne and Cudek (1993) argued that values ranging from 0.05 to 0.08 are indicative of a good adequacy of the model.

Internal consistencies for the scales composing of the best fitting model were computed using Cronbach's coefficient alpha for each factor.

### Measures

#### Capacity to love inventory (CTL-I)

The Capacity to Love Inventory (CTL-I) (Kapusta et al., 2018), translated through the back translation process (Van de Vijver and Leung, 1997), is a 41 items questionnaire rated on a 4 point Likert Scale and composed of six dimensions: interest in the life project of the other (INT), basic trust (BRT), gratitude (GRT), common ego ideal (CEI), permanence of sexual passion (PSP) and loss and mourning (LOM). In the original version, Cronbach's alpha of the total scale is 0.90, whereas in the current study is 0.94; for the subscales values are 0.84 for Interest, 0.83 for Trust, 0.85 for Humility, 0.87 for Ego, 0.84 for Passion, and 0.85 for Acceptance.

#### Romance qualities scale (RQS)

The Romance Qualities Scale (RQS) (Ponti et al., 2010) is a self-report questionnaire which measures the qualitative aspects of romantic relationships. It consists of 22 items, evaluated on a 5-point Likert Scale, developed in five dimensions: Conflict, Companionship, Help, Security, Closeness. The internal consistency coefficient for the five subscales were: 0.74 and 0.75 for Conflict; 0.61 and 0.62 for Companionship; 0.82 and 0.84 for Help; 0.69 and 0.72 for Security; 0.74 and.78 for Closeness. In the present study, Cronbach's alphas were: 0.74 for Conflict; 0.49 for Companionship, 0.88 for Help, 0.73 for Security, 0.86 for Closeness, and 0.85 for the total scale.

#### Beck depression inventory (BDI-II)

The Beck Depression Inventory (BDI-II) is a 21 item self-report inventory that assess the presence and severity of depressive symptoms, according to DSM-IV (American Psychiatric Association, 1994) criteria. Statements regarding some feelings are rated on a 4-point Likert scale, based on the severity of depressive symptoms. The literature supported the inventory's psychometric properties in clinical and non-clinical samples (e.g., Arbisi, 2001; Balsamo and Saggino, 2007). In the current study, Cronbach's alpha was 0.88.

#### Socio-sexual orientation inventory-revised (SOI-R)

The Socio-sexual Orientation Inventory-Revised (SOI-R), in its Italian translation (Penke and Asendorpf, 2008), measures socio-sexual orientation assessing three facets of socio-sexuality: Past Behaviour as number of casual and changing sex partners, explicit Attitude toward uncommitted sex, sexual Desire. It is composed by nine self-report items, with a five-point Likert scale. Cronbach's alpha is high for Behaviour facet (0.84f−0.85m), Attitude (0.83f−0.87m), Desire (0.85 f−0.86m), Total (0.83). In the present study the Cronbach's alpha: 0.52 for Behaviour, 0.51 for Attitude, 0.80 for Desire, and 0.72 for the total scale.

#### Pathological narcissism inventory (PNI)

The Pathological Narcissism Inventory (PNI) (Pincus et al., 2009) is composed by 52-items rated on a six-point Likert scale. Items were translated into Italian according to procedures of back translation. It assesses the dimensions of narcissistic grandiosity and narcissistic vulnerability. In the present study the internal consistency coefficients were 0.74 for narcissistic grandiosity, 0.82 for narcissistic vulnerability, and 0.94 for the total scale.

#### Narcissistic personality inventory (NPI)

The Narcissistic Personality Inventory (NPI), in its Italian validation (Fossati et al., 2008) is characterized by 40 items and 7 sub-scales: Authorities, Exhibitionism, Superiority, Feeling in Law, Manipulation, Self-sufficiency, and Vanity. The internal consistency coefficients for the total of NPI (0.83) and for the sub-scale Authority (0.73) is good. In the present study the Cronbach's alpha is 0.69 for Authorities, 0.67 for Exhibitionism, 0.52 for Superiority, 0.46 for Feeling in Law, 0.50 for Manipulation, 0.38 for Self-sufficiency, 0.60 for Vanity, and 0.81 for the total scale.

## Results

We tested a theory driven model by means of a CFA: a six-factor model with 41 items and scales being allowed to correlate with each other (Tables 2, 3).

**Table 2:** Factor loadings of 41-item CTL-I.

**#**	**Item**	**INT**	**BTR**	**GRT**	**CEI**	**PSP**	**LOM**
1	È importante conoscere il progetto di vita del mio partner.	0.702					
2	Condivido i progetti di vita con il mio partner.	0.641					
3	Sono felice di condividere i successi del mio partner.	0.875					
4	Mi arricchisce vedere la crescita personale del mio partner.	0.836					
5	Se il mio partner è infelice, mi sento triste.	0.528					
6	Capisco come essere comprensivo con il mio partner.	0.549					
7	Mi annoio spesso con il mio partner^*^	0.555					
8	Conto sul fatto che il mio partner sia comprensivo nei miei confronti se necessario.		0.572				
9	Il mio partner conosce le mie debolezze, i miei conflitti interiori e i miei problemi.		0.718				
10	Posso esprimere apertamente al mio partner sentimenti e bisogni personali.		0.793				
11	Conto sul fatto che il mio partner mi sostenga emotivamente nelle situazioni di incertezza.		0.751				
12	Sento di essere onesto con il mio partner.		0.591				
13	Nascondo dei segreti al mio partner^*^		0.452				
14	Posso confessare le mie debolezze al mio partner.		0.747				
15	A volte sento che ci sono dei limiti nei miei rapporti che non riesco a superare, nonostante gli sforzi fatti.^*^		0.220				
16	Sono a mio agio con il mio partner e di solito mi sento al sicuro.		0.698				
17	Avverto un senso di gratitudine per il fatto che il mio partner ci sia.			0.699			
18	Sono grato per l'amore ricevuto.			0.688			
19	Anche se siamo lontani per molto tempo continuo a sentirmi legato al mio partner.			0.698			
20	Riconosco di aver bisogno del mio partner.			0.693			
21	Mi piace confortare il mio partner.			0.640			
22	Mi piace prendermi cura del mio partner, quando ha bisogno del mio aiuto.			0.643			
23	Condivido le responsabilità del quotidiano per sollevare il mio partner.			0.679			
24	Mi impegno nelle relazioni.				0.706		
25	Con il mio partner cerchiamo di lavorare sul nostro rapporto.				0.661		
26	Ho rispetto per la personalità e i valori del mio partner.				0.748		
27	Adoro guardare i gesti e le reazioni del mio partner.				0.580		
28	Mi sento impegnato nella nostra vita comune.				0.617		
29	Cerco possibili punti di incontro nei casi di conflitti e prospettive differenti.				0.788		
30	Dico spesso al mio partner che lo amo.				0.767		
31	Mi sento profondamente legato al mio partner.				0.616		
32	In un rapporto a lungo termine ad un certo punto sopraggiunge la noia nella sfera sessuale. ^*^					0.836	
33	Col passare del tempo diminuisce il desiderio sessuale. ^*^					0.859	
34	È difficile accettare che la persona che amo non corrisponda il mio amore. ^*^						0.606
35	Se un rapporto finisce, tendo ad attribuire la colpa al mio ex-partner.^*^						0.682
36	Provo desideri di vendetta se il mio partner mi lascia. ^*^						0.683
37	Spesso ho difficoltà ad accettare la fine delle mie relazioni. ^*^						0.693
38	Sono spesso geloso. ^*^						0.577
39	Provo spesso sensi di colpa dopo una separazione.^*^						0.603
40	Se il partner mi ha abbandonato tendo a svalutarmi. ^*^						0.673
41	È difficile essere coinvolto nuovamente in un rapporto dopo la fine di una relazione. ^*^						0.581

* *Reverse-coded Item. INT, Interest; BTR, Basic Trust; GRT, Gratitude; CEI, Common Ego Ideal; PSP, Permanence of Sexual Passion; LOM, Loss and Mourning*.

**Table 3:** Correlation between CTL-Ifactors.

	**INT**	**BTR**	**GRT**	**CEI**	**PSP**	**LOM**
INT	-	0.771^**^	0.657^**^	0.667^**^	0.151^**^	0.385^**^
BTR		-	0.689^**^	0.668^**^	0.184^**^	0.275^**^
GRT			-	0.939^**^	0.303^**^	0.116^*^
CEI				-	0.349 ^**^	0.098^*^
PSP					-	0.244^**^
LOM						-

** *p < 0.01*,

* *p < 0.05. INT, Interest; BTR, Basic Trust; GRT, Gratitude; CEI, Common Ego Ideal; PSP, Permanence of Sexual Passion; LOM, Loss and Mourning*.

Fit indices were: *chi2*/degrees of freedom = 3.40 (2598.870/764), *SRMR* = 0.053, *RMSE* = 0.057 (90% *CI* 0.055–0.060).

Based on the results of the χ2 statistic, lack of overall fit for the model tested (*p* < 0.001) was shown, probably due to the sensitivity of this statistic to large sample sizes (Hu and Bentler, 1998; Kahn, 2006). In fact chi-square is highly sensitive to sample size: as the size of the sample increases, absolute differences become a smaller and smaller proportion of the expected value. The larger the sample, the larger and significant will be the chi squares, even with very small discrepancies among implied and obtained covariance matrices. On the other hand, samples of reduced size may be too prone to accept poor models (Type II error).

According to other goodness-of fit indices, that are RMSEA and SRMR, a good adequacy of the model was shown (Browne and Cudek, 1993; Hu and Bentler, 1999).

Results were very similar to those obtained by Kapusta et al. (2018) in the original version of CTL-I: *chi2*/degrees of freedom = 3.13 (2598.870/764), *SRMR* = 0.060, *RMSEA* = 0.062 (90% *CI* 0.060–0.065).

In order to investigate the potential associations among the six CTL-I subscales and other related measures, Pearson correlational analyses were computed (Table 4).

**Table 4:** Pearson correlations between CTL-I Italia version and concurrent measures.

	**CTL-I**						
	**Interest**	**Basic trust**	**Gratitude**	**Common ego ideal**	**Permanence of sexual passion**	**Loss and mourning**	**Total scale**
RQS	0.154^**^	0.247^**^	0.259^**^	0.198^**^	−0.209^**^	−0.072	0.196^**^
Conflict	−0.098	−0.090	−0.126^*^	−0.176	0.078	0.213^**^	0.066
Companionship	0.174^**^	0.219^**^	0.272^**^	0.246^**^	−0.246^**^	−0.134^*^	0.188^**^
Help	0.165^**^	0.274^**^	0.298^**^	0.235^**^	−0.216^**^	−0.119^*^	0.214^**^
Security	0.165^**^	0.234^**^	0.221^**^	0.218^**^	−0.175^**^	−0.182^**^	0.160^**^
Closeness	0.146^**^	0.216^**^	0.277^**^	0.231^**^	−0.210^**^	−0.032	0.214^**^
SOI–R	−0.125	−0.019	−0.168^**^	−0.111^*^	0.139^*^	−0.011	−0.105
Behaviour facet	−0.96	−0.023	−0.100	−0.051	−0.059	−0.050	−0.050
Attitude	−0.142^*^	−0.035	−0.122^*^	−0.106	0.093	0.062	−0.118^*^
Desire	−0.063	−0.006	−0.164^**^	−0.092	0.150^**^	0.015	−0.070
PNI	−0.041	0.012	0.009	−0.051	0.086	0.555^*^	0.164^**^
Narcissistic grandiosity	0.014	0.094	0.041	−0.030	0.029	0.418^**^	0.165^**^
Narcissistic vulnerability	−0.060	−0.026	−0.003	−0.050	0.101	0.563^**^	0.150^**^
NPI	−0.038	−0.011	−0.075	−0.061	−0.002	0.072	−0.031
Authorities	−0.015	0.055	−0.056	−0.071	−0.009	0.028	0.020
Exhibitionism	−0.072	−0.099	−0.121^*^	−0.132^*^	0.025	0.182^**^	0.063
Superiority	−0.013	0.001	−0.001	−0.006	0.054	0.112^*^	−0.030
Feeling in Law	−0.095	0.049	−0.154^**^	−0.124^*^	0.070	0.264^**^	0.003
Manipulation	0.027	0.034	−0.022	−0.029	0.018	0.003	0.002
Self-sufficiency	0.043	−0.007	−0.018	0.072	−0.111	−0.195^**^	−0.049
Vanity	−0.030	−0.058	−0.057	−0.074	−0.035	0.056	−0.051
BDI–II	−0.074	−0.100	−0.131^*^	−0.096	−0.137^*^	0.347^**^	0.011

** *p < 0.01*,

* *p < 0.05 RQS, Romance Qualities Scale; SOI-R, Socio-sexual Orientation Inventory-Revised; PNI, Pathological Narcissism Inventory; NPI, Narcissistic Personality Inventory; BDI-II, Beck Depression Inventory*.

The subscales of the CTL-I show specific associations with the external correlated measures.

CTL-I *Interest* and CTL-I *Basic Trust* were positively correlated with the subscales of RQS related to Companionship, Help, Security and Closeness.

CTL-I *Gratitude* is positively correlated with the subscales of RQS Companionship, Help, Security, Closeness, and negatively correlated with the Conflict subscale of RQS and the Attitude and Desire subscales of SOI-R, and subscale Exhibitionism and Feeling in love of NPI.

*CTL Common Ego Ideal* is positively correlated with the Companionship, Help, Security, Closeness subscale of RQS and negatively correlated with Exhibitionism and Feeling in love of NPI.

CTL-I *Permanence of Sexual Passion* is negatively correlated with Companionship, Help, Security, Closeness subscales of RQS and depression (BDI-II) but positively correlates with Desire subscale of SOI-R.

CTL-I *Loss and Mourning* is positively correlated with Conflict subscale of RQS, with Narcissistic grandiosity and Narcissistic vulnerability of PNI, with the Exhibitionism, Superiority, Feeling in Law, Self-sufficiency subscale of PNI, and with depression of BDI-II. CTL-I *Loss and Mourning* is negatively correlated with Companionship, Help, Security, of RQS and Self-sufficiency subscale of NPI.

## Discussion

The present study showed that the Italian version of the CTL-I exhibited a clear factor structure and good psychometric properties. Indeed, Cronbach's alphas for all subscales were good, as well as its convergent validity.

The CFS's results showed a fully satisfactory fit. The dimensions emerging from this analysis showed superimposable to the ones obtained by Kapusta et al. (2018) in their original study. Thus, it is possible to conclude that the Italian CTL-I displayed a good psychometric functioning, similar to the one shown by the inventory's original version.

From our analysis it emerged that the CTL-I measures the capacity to love in a romantic relationship, as demonstrated by the strong positive and significant correlation with the RQS. Concerning the capability of measuring dimensions of psychopathology, the dimension of *CTL-I Loss and Mourning* strongly showed a moderate negative correlation with the subscales of the PNI and with the BDI-II, showing the possibility that capacity to love displays a protective function against the emergence of some symptomatic expressions.

Although we did not administer to a clinical sample, we believe that the instrument has some important clinical implications. First of all, the negative correlation of the CTL-I with the clinical scales should confirm the important diagnostic potentialities of the capacity to love (Gargiulo et al., 2014).

Moreover, considering the need to develop dynamic approaches to study the psychotherapeutic processes and outcomes (Bateman and Fonagy, 2012; Gelo and Salvatore, 2016; Esposito et al., 2017), the CTL-I could be a useful instrument for monitoring the efficacy of clinical intervention or their process-outcome connections. From a perspective which considers therapeutic change as an integration between the reorganization of affects and a different representation of objects relationships (Kernberg, 1993; De Luca Picione et al., 2018), we believe that the scale could catch the processes of therapeutic change of a clinical intervention which should aim at reorganizing the psychic mental set of a patient as a function of new relationship modalities.

Regarding the level of the clinical relationship, we believe that the CTL-I could be a useful indicator in order to comprehend the quality of the transfert.

Finally, due to its flexibility we believe that the instrument could be also able to individuate risk and protective dimensions in non-psychotherapeutic clinical contexts (Carlino and Margherita, 2016; Margherita et al., 2017; Tessitore and Margherita, 2017).

This study is not free from limitations. One of the limitations of the study is the small number of men in the samples; this could be explained hypothesizing that men may be less interested in romantic relationships compared to women (Fraley et al., 2011). Future investigations basedfocused on gender differences and couple relationships are needed. Another limitation regards the recruitment of the sample, unbalanced between online and offline. Moreover, considering the capacity to love as a non-stable characteristic, future research also needs to consider it depending on the relationships.

## Ethics statement

This study was carried out in accordance with the recommendations of the ethical guidelines for research draft by the Italian Psychologists Association and the National Psychologists Council. The ethical guidelines of Helsinki Declaration were followed and participants were informed about the confidentiality of the data and the treatment. The protocol was approved by the Local Ethical Committee for research in Psychology.

## Author contributions

All the authors listed have made a substantial contribution to the work. GM developed the theoretical framework of the present study, designed the research project and contributed to the scientific supervision of the study. AG contributed to the methodological approach, performed the translation procedures of the instrument and the data collection and wrote the manuscript. GT performed all the analysis and designed tables and figures. FT specifically wrote the Introduction and revised the manuscript. NK developed the theoretical framework of the whole project, operationalized the construct of the capacity to love and contributed to the scientific supervision of the whole work. All authors discussed the results, commented the manuscript and gave the final approval of the work.

### Conflict of interest statement

The authors declare that the research was conducted in the absence of any commercial or financial relationships that could be construed as a potential conflict of interest.
